# ECONOMIC BOOM AND FINANCIAL RECESSION—WHAT ARE THE EFFECTS ON RELOCATION PATTERNS FOR OLDER ADULTS?

**DOI:** 10.1093/geroni/igad104.2503

**Published:** 2023-12-21

**Authors:** David Dahlgren, Asa Hansson, Susanne Iwarsson, Steven Schmidt, Maya Kylén

**Affiliations:** Lund University, Lund, Skane Lan, Sweden; Lund University, Lund, Skane Lan, Sweden; Lund University, Lund, Skane Lan, Sweden; Lund University, Lund, Skane Lan, Sweden; Lund University, Lund, Skane Lan, Sweden

## Abstract

While previous research shows that older adults choose to relocate to a different home for numerous reasons (e.g., changes in the household composition, health decline), little is known about the influence of economic factors. The economic situation on aggregated level can influence the individual in more ways than by consumer demand and income, for example, financial crisis, fluctuating housing prices, and more general uncertainties about the future. Representing a novel approach, the aim of this study was to investigate the impact of macroeconomic events on relocation rates among older adults in Sweden. We used high quality panel data from Swedish registers, based on a random sample made 1994 with 300,000 individuals. Data was analyzed from 1970-2016 using a repeated cross-sectional design. Preliminary results show that the number of relocations was volatile the first year of a financial crisis. Depending on the characteristics of the crisis, the number of relocations decreased or increased; crises that are less connected to the property market had a reduction in number of relocations. This effect was observed for 1-2 years and stabilized to “normal levels” in the subsequent years. Especially high volatility was observed for the older age groups (75-84 and 85-90), which could be because they have a shorter time horizon for investment planning and great need to transfer housing wealth to liquidity. Knowledge in this field enables policymakers to plan housing investment, and negative shocks to the economy may lead to short term impacts on the housing market for older adults.

